# The Recognition of N-Glycans by the Lectin ArtinM Mediates Cell Death of a Human Myeloid Leukemia Cell Line

**DOI:** 10.1371/journal.pone.0027892

**Published:** 2011-11-23

**Authors:** Fernanda Caroline Carvalho, Sandro Gomes Soares, Mirela Barros Tamarozzi, Eduardo Magalhães Rego, Maria-Cristina Roque-Barreira

**Affiliations:** 1 Departamento de Biologia Celular e Molecular e Bioagentes Patogênicos, Faculdade de Medicina de Ribeirão Preto, Universidade de São Paulo, Ribeirão Preto, São Paulo, Brasil; 2 Invent Biotecnologia Ltda, Ribeirão Preto, São Paulo, Brasil; 3 Departamento de Clínica Médica, Faculdade de Medicina de Ribeirão Preto, Universidade de São Paulo, Ribeirão Preto, São Paulo, Brasil; Karolinska Institutet, Sweden

## Abstract

ArtinM, a d-mannose-binding lectin from *Artocarpus heterophyllus* (jackfruit), interacts with N-glycosylated receptors on the surface of several cells of hematopoietic origin, triggering cell migration, degranulation, and cytokine release. Because malignant transformation is often associated with altered expression of cell surface glycans, we evaluated the interaction of ArtinM with human myelocytic leukemia cells and investigated cellular responses to lectin binding. The intensity of ArtinM binding varied across 3 leukemia cell lines: NB4>K562>U937. The binding, which was directly related to cell growth suppression, was inhibited in the presence of Manα1-3(Manα1-6)Manβ1, and was reverted in underglycosylated NB4 cells. ArtinM interaction with NB4 cells induced cell death (IC_50_ = 10 µg/mL), as indicated by cell surface exposure of phosphatidylserine and disruption of mitochondrial membrane potential unassociated with caspase activation or DNA fragmentation. Moreover, ArtinM treatment of NB4 cells strongly induced reactive oxygen species generation and autophagy, as indicated by the detection of acidic vesicular organelles in the treated cells. NB4 cell death was attributed to ArtinM recognition of the trimannosyl core of N-glycans containing a ß1,6-GlcNAc branch linked to α1,6-mannose. This modification correlated with higher levels of N-acetylglucosaminyltransferase V transcripts in NB4 cells than in K562 or U937 cells. Our results provide new insights into the potential of N-glycans containing a β1,6-GlcNAc branch linked to α1,6-mannose as a novel target for anti-leukemia treatment.

## Introduction

Aberrant glycosylation of cell-surface glycoconjugates is a universal feature of cancer cells [1]. These alterations may be instrumental in the failure of intercellular contact and communication [2] and in the invasive and infiltrative properties of cancerous cells. Several studies have evaluated lectin binding to malignant cells [3]–[6]. The recognition of altered glycosylation in cancer cells by specific lectins has aided the assessment of cancer disease status [7], [8].

Lectins are carbohydrate-binding proteins or glycoproteins of non-immune origin that recognize and reversibly bind to glycans without altering their covalent structure. Plant lectins are important tools in cell biology and immunology, with potential for clinical application [8], [9]. Lectins can identify glycan determinants that are markers of clinical interest and may possess anti-tumor and anticarcinogenic properties that could be useful in the development of cancer therapeutics. Several studies have suggested that lectins can induce apoptosis in several human cancer cell lines [10]–[12].

ArtinM (also known as KM+ and Artocarpin) [13], a lectin from *Artocarpus heterophyllus*, binds d-mannose and exhibits high specificity for the trimannoside Manα1-3[Manα1-6]Man, present in the core of N-glycans [14]. ArtinM possesses many relevant biological properties. It acts on neutrophils, inducing haptotactic migration and phenotypic and functional changes, which include intracellular tyrosine phosphorylation, shedding of l-selectin, release of inflammatory mediators, phagocytic and cell-killing activities, and increased expression of TLR2 [15], [16]. Furthermore, an amplification loop for *in vivo* ArtinM inflammatory activity is provided by induction of mast cell degranulation [17]. ArtinM stimulates macrophage and dendritic cells to release IL-12, thereby establishing *in vivo* Th1 immunity and conferring protection against several intracellular pathogens [18]–[20]. ArtinM also accelerates wound healing and epithelial tissue regeneration [33] Pinto-da-Silva LL, Panunto-Castelo A, de Souza Goldman MH, Roque Barreira MC, de-Oliveira RS, Dias-Baruffi M, Blanco de Molfetta Machado. J. WIPO, Patent WO2004100861; 2004. [21].

Previous data on ArtinM activity on cells of hematopoietic origin led us to investigate the direct effect of ArtinM on leukemia cells.

## Results

### ArtinM distinctly interacts with leukemia cells and inhibits growth of NB4 cells

**Figure 1:**
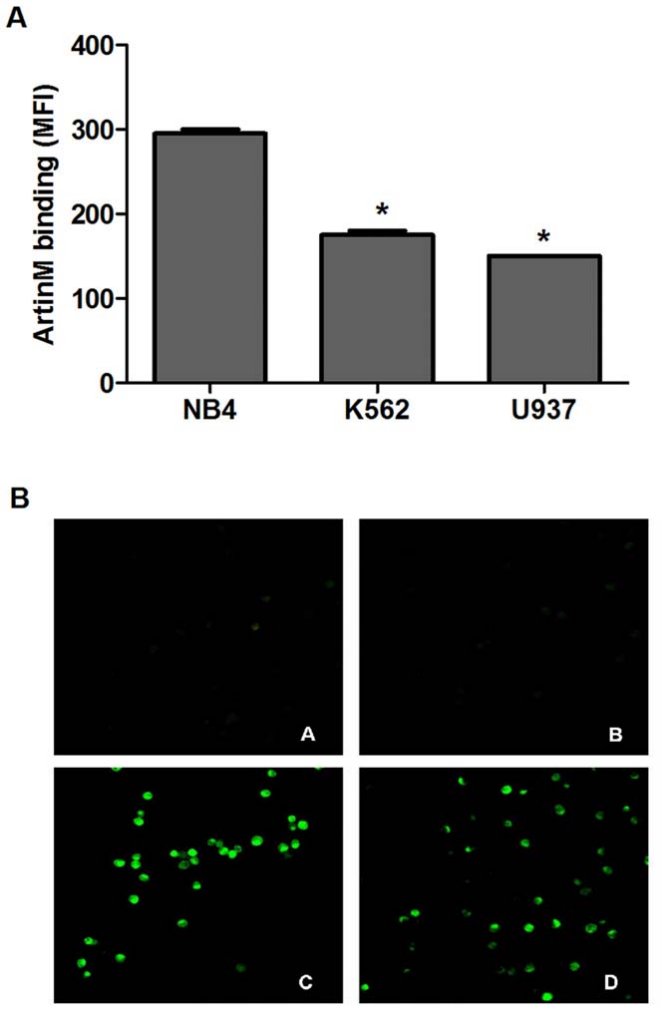
ArtinM interacts with leukemia cell lines. **A**) ArtinM binding to NB4, K562, and U937 cells: cells were fixed and incubated for 30 min with biotinyl-ArtinM/streptavidin-FITC (5 µg/mL). Lectin binding to the cell surface was detected by flow cytometry and expressed as mean fluorescence intensity (MFI). **B**) NB4 cells adhered to Biobond-coated coverslips were incubated at 4°C for 60 min with biotinyl-ArtinM (5 µg/mL) (panel C) or PBS (panel A). For inhibition assays, biotinyl-ArtinM was pre-incubated at room temperature for 60 min with 10 µM Manα1-3[Manα1-6]Man (panel B) or 200 mM d-galactose (panel D), and then incubated with NB4 cells. After washing and incubation with streptavidin-FITC, cells were fixed and examined by fluorescence microscopy. Magnification = 40×. The result shown are representative of 3 independent experiments and are expressed as mean ± SD, * p<0.05 (Tukey's test).

Malignant transformation is accompanied by the modification of surface glycans, which can become targets for lectin recognition [1], [8]. We used flow cytometry to evaluate ArtinM binding to 3 different leukemia cell lines. The level of cell staining indicative of ArtinM binding to each cell line is shown in Figure 1A. The fluorescence intensity in NB4 cells was at least 30% higher than in K562 and U937 cells, despite the ability of ArtinM lectin to bind more than 95% of cells in each cell line (data not shown). Fluorescence microscopy confirmed ArtinM binding to NB4 cells (Fig. 1C); this binding was completely inhibited by pre-incubation with 10 µM Manα1-3[Manα1-6]Man (panel B), but not with 200 mM d-galactose (panel D), indicating that NB4 cell recognition by ArtinM is mediated by its carbohydrate recognition domain.

**Figure 2:**
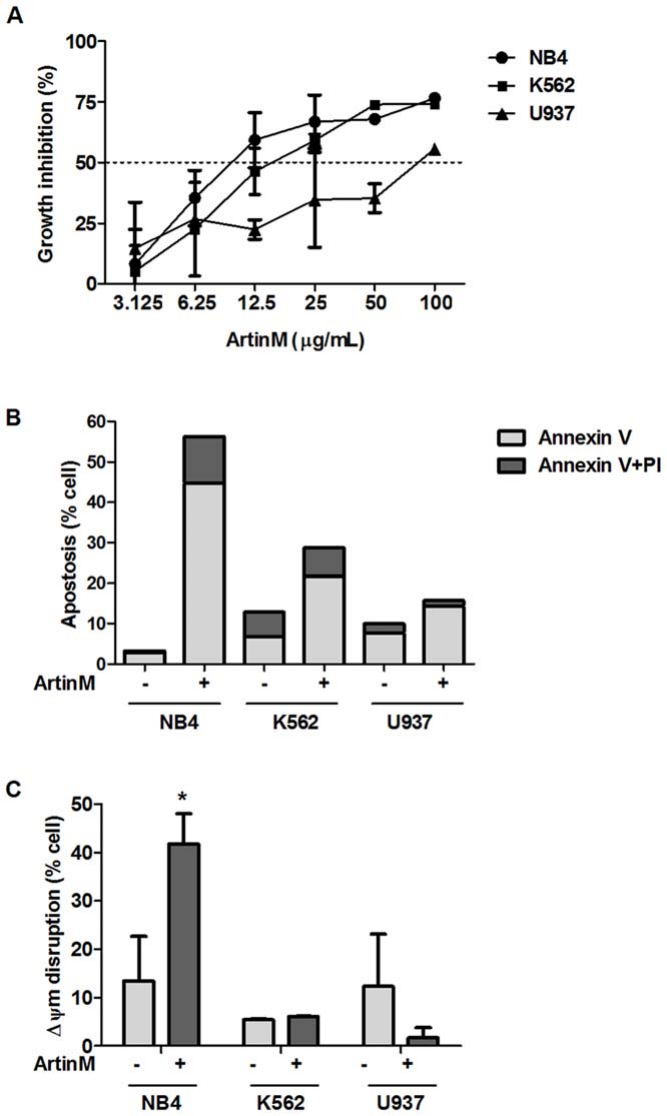
ArtinM inhibits cell growth inhibition and pro-apoptotic effect on NB4 cells. **A**) NB4, K562, and U937 cells were cultured for 48 h in the presence or of ArtinM (3.125 to 100 µg/mL). Cell growth was evaluated by MTT assay. The curves show the ArtinM inhibition of cell growth relative to the growth of untreated cells. Each point was obtained from a triplicate assay, and the results shown are representative of 3 independent experiments. The dotted horizontal line indicates the ArtinM concentration necessary for 50% growth inhibition (IC_50_). **B**) NB4, K562, and U937 cells were cultured for 48 h in the presence or absence of ArtinM (10 µg/mL, IC_50_ for NB4). Cells were stained with FITC-Annexin V and propidium iodide (PI) to characterize apoptosis and to distinguish it from necrosis. Bars represent the proportion of Annexin V and Annexin V/PI-stained cells. Results represent 3 different experiments. **C**) Disruption of mitochondrial membrane potential (mΔΨ) of ArtinM-treated cells was assessed by flow cytometry after staining with JC-1.The bars represent the percentage of cells with mΔΨ disruption. The result shown are representative of 3 independent experiments and are expressed as mean ± SD, * p<0.05 (Tukey's test).

Considering that lectin interactions with tumor cells can trigger biochemical responses [9], we investigated whether various levels of ArtinM binding to the surface of leukemia cells could affect their growth. We used MTT assays to determine cell viability and generated growth inhibition curves for different ArtinM concentrations, as shown in Figure 2A. We thus determined the ArtinM concentrations that inhibit 50% of cell growth (IC_50_). NB4 and K562 cells were more sensitive to ArtinM inhibition, displaying IC_50_ of 10 (±1) and 14 (±1) µg/mL, respectively, while U937 cells exhibited an IC_50_ of 84 (±1,5) µg/mL.

We performed a more detailed study to understand the effects of ArtinM on leukemia cells. NB4, K562, and U937 leukemia cells were cultured with ArtinM at 10 µg/mL, the IC_50_ for NB4, and after 48 h, the cells were analyzed by flow cytometry for Annexin V staining and PI incorporation. In NB4 cells, ArtinM induced pronounced surface exposition of phosphatidylserine, as revealed by Annexin V binding and minor PI incorporation, suggesting the occurrence of apoptosis. In K562 and U937 cells, ArtinM provoked lower levels of Annexin V binding and PI staining (Fig. 2B).

The mitochondrial transmembrane electrical potential of leukemia cells, following 48 h incubation with 10 µg/mL ArtinM, was evaluated by JC-1 dye. ArtinM treatment promoted the disruption of mitochondrial transmembrane electrical potential in NB4 cells (Fig. 2C). The other tested cell lines (K562 and U937) were resistant to ArtinM treatment (10 µg/mL).

### Non-differentiation and caspase-independent cell death could be related to autophagy under ArtinM treatment

**Figure 3:**
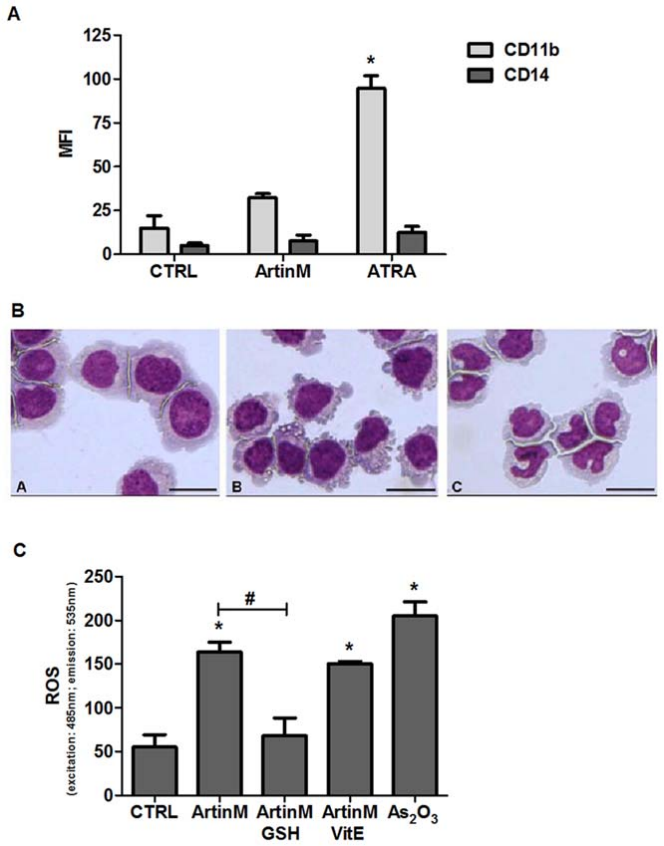
ArtinM does not induce NB4 differentiation but promotes ROS generation. NB4 cells were cultured for 48 h with ArtinM (10 µg/mL) or medium (CTRL), and then analyzed for 3 parameters. ATRA (1 µg/mL) or As_2_O_3_ (1 µM) were used as positive controls. **A**) Expression of CD11b and CD14 was evaluated by flow cytometry and expressed as mean fluorescence intensity (MFI). **B**) Morphology of NB4 cells was evaluated by optical microscopy of HEMA-stained cytospin preparations. Panel A: NB4 cells; Panel B: ArtinM-treated NB4 cells; Panel C: ATRA-treated NB4 cells. Bar = 10 µm. **C**) ROS production was evaluated by measuring the oxidative conversion of DCFH-DA to DCF in a fluorospectro-photometer. The effect of reduced glutathione (GSH) or α-tocopherol acetate (VitE) addition to ArtinM-stimulated cultures was also assayed. The results are expressed as optical density (OD) at excitation and emission wavelengths of 485 and 535 nm, respectively. The result shown are representative of 3 independent experiments and are expressed as mean ± SD, * p<0.05 (Tukey's test); # p<0.05 (ArtinM×ArtinM GSH, Tukey's test).

Since augmented cell death rates could be caused by induction of leukemia cell differentiation, as often happens following all-trans retinoic acid (ATRA) therapeutic administration [22], we investigated whether induction of NB4 cell differentiation could account for the observed effect of ArtinM. As shown in Figure 3A, ArtinM did not induce NB4 cell differentiation, as demonstrated by levels of CD11b surface expression, which are 3 times lower after ArtinM treatment than after ATRA treatment. ArtinM-treated NB4 cells did not exhibit the multilobulated nucleus that characterizes differentiated granulocytes, a feature that was observed in ATRA-treated cells (Fig. 3B). Cytoplasmic vacuolization was also observed in ArtinM-treated NB4 cells. Neither lower doses of ArtinM nor increased length of exposure changed the non-differentiated status of the cells (data not shown).

The absence of cell differentiation and the detection of mitochondrial membrane depolarization led us to investigate the occurrence of oxidative stress. ArtinM-stimulated NB4 cells generate high levels of reactive oxygen species (ROS), similar to those observed in the positive control (arsenic trioxide, As_2_O_3_) (Fig. 3C). The augmented ROS production was inhibited to basal levels in the presence of reduced glutathione (GSH). In contrast, α-tocopherol acetate (vitamin E) had no effect on the cellular response to ArtinM.

**Figure 4:**
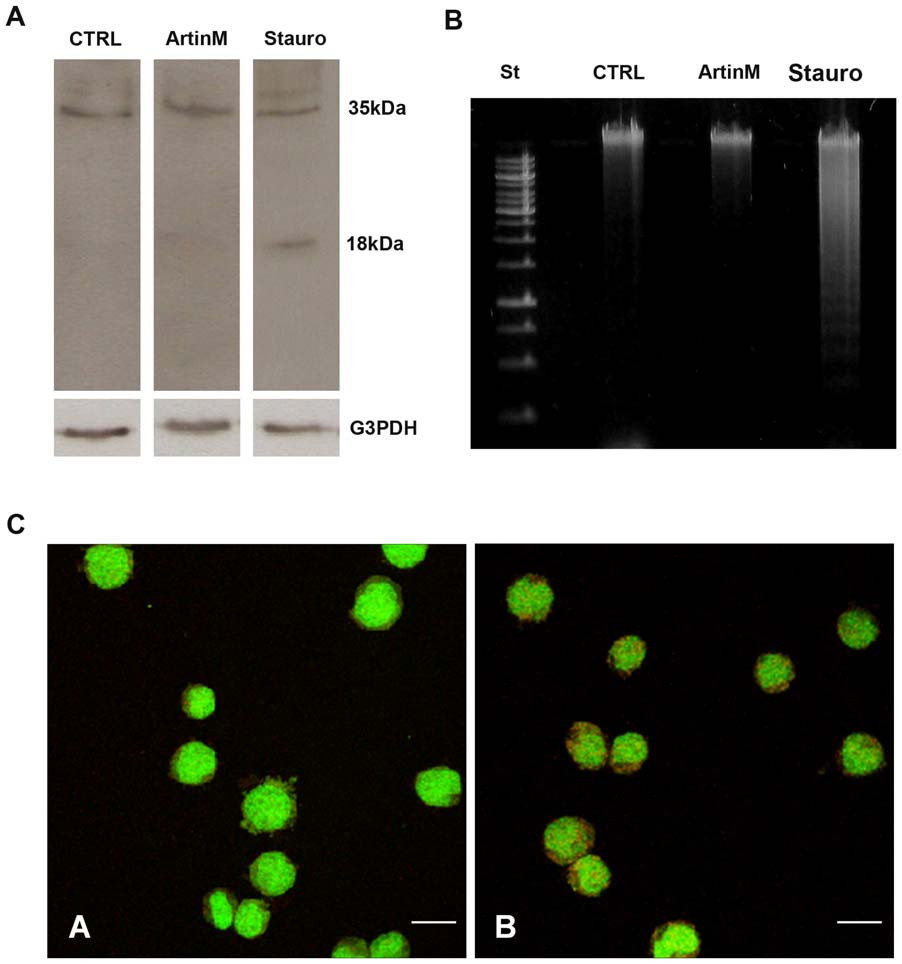
Autophagy accounts for the caspase-independent mechanism of NB4 cell death induced by ArtinM. NB4 cells were cultured with ArtinM (10 µg/mL) for 48 h or with Staurosporine (Stauro, 5 µM) for 4 h. **A**) Caspase-3 activation, manifested by cleavage of their precursor forms, was analyzed by Western blotting of RIPA cell lysates (100 µg protein) performed under reducing conditions. **B**) Fragmentation of genomic DNA from NB4 cells was evaluated by electrophoresis on 1% agarose gel followed by ethidium bromide visualization. St: standard markers. **C**) Acidic autophagic vacuoles in NB4 cells were detected through staining with 10 µg/mL acridine orange in serum-free medium. Fluorescent micrographs show that the cytoplasm and nucleus of stained cells fluoresced bright green, whereas the acidic autophagic vacuoles fluoresced bright red. Bar = 10 µm.

To assess whether the signaling event promoted by ArtinM led to apoptosis, we investigated the involvement of caspase activity in ArtinM-mediated cell growth inhibition as compared to staurosporine-mediated apoptosis. Caspase-3 remained in its precursor form (not activated, 35 kDa) in ArtinM-treated cells, as observed by Western blot analysis of cell lysates (Fig. 4A). In staurosporine-treated cell lysates (positive control), the active caspase-3 fragment (18 kDa) was detected. Analysis of low-molecular-weight DNA extracted from ArtinM-treated cells did not show the characteristic ladder pattern resulting from internucleosomal cleavage of genomic DNA (Fig. 4B). These results suggest that ArtinM promotes caspase-independent cell death in NB4 cells.

Since intense cytoplasm vacuolization (Fig. 3B, panel B) was verified in NB4 cells treated with ArtinM (10 µg/mL for 48 h), we hypothesized that the lectin could induce autophagy-mediated cell death. The characteristic formation of acidic vesicular organelles (AVOs), stained with aggregated acridine orange, was examined by confocal microscopy. We found a pronounced increase in AVOs in NB4 cells incubated with ArtinM (Fig. 4C, panel B) in comparison to untreated cells (Fig. 4C, panel A).

### The action of ArtinM on NB4 cells is mediated by recognition of N-glycans

**Figure 5:**
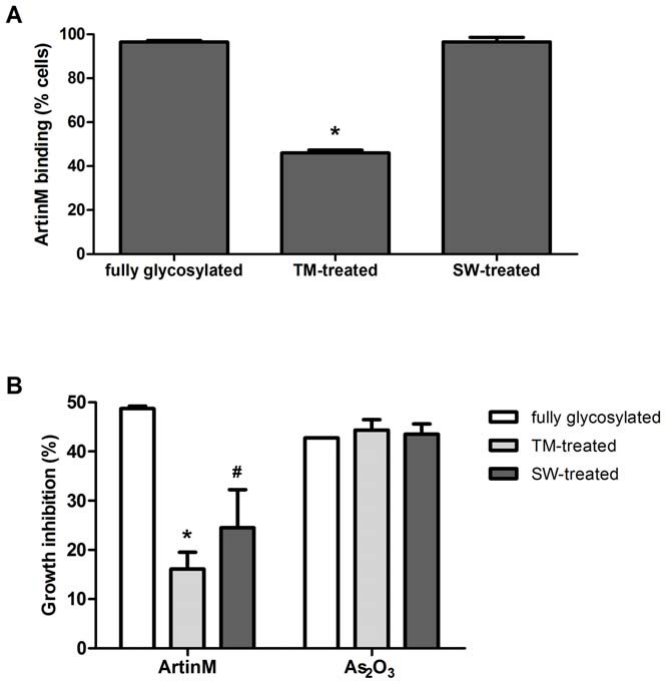
N-Glycan recognition by ArtinM accounts for NB4 growth inhibition. **A**) ArtinM binding to NB4 cells pre-treated for 24 h with tunicamycin (TM, 5 µg/mL) or swainsonine (SW, 5 µg/mL) was analyzed by flow cytometry. Fully glycosylated: untreated NB4 cells. **B**) Growth inhibition induced by ArtinM (10 µg/mL) in fully glycosylated cells, and in TM- or SW-treated cells. Growth rate was measured by MTT assay. Bars represent cell growth inhibition by ArtinM relative to the growth of untreated cells. As_2_O_3_ (1 µM) was used as a positive control for cell death. The results shown are representative of 3 independent experiments and are expressed as mean ± SD, * p<0.05 (Tukey's test) and ^#^ p<0.05 (Student's *t*-test).

In order to characterize the relationship between ArtinM N-glycan recognition and NB4 cell growth inhibition, we added tunicamycin (TM, 5 µg/mL) or swainsonine (SW, 5 µg/mL) to NB4 cells that were subsequently stimulated with ArtinM after 24 h. TM and SW alone resulted in NB4 growth inhibition rates of 30% and 10%, respectively, the viable cell was tested to ArtinM binding and ArtinM growth inhibition. In comparison with fully glycosylated cells (untreated cells), ArtinM binding of TM-treated NB4 cells was strongly inhibited, whereas ArtinM binding of SW-treated cells was preserved (Fig. 5A).

We next evaluated whether TM- or SW-treated NB4 cells were responsive to ArtinM cell growth inhibition. Indeed, ArtinM inhibition of cell growth reached 60% in TM-treated cells and 45% in SW-treated cells, as compared with fully glycosylated cells (Fig. 5B). In contrast, the inhibition of NB4 cell growth by As_2_O_3_ (positive control) was not affected by the glycosylation status. These results point to the importance of Manα1-6 elongation in effective ArtinM response.

**Figure 6:**
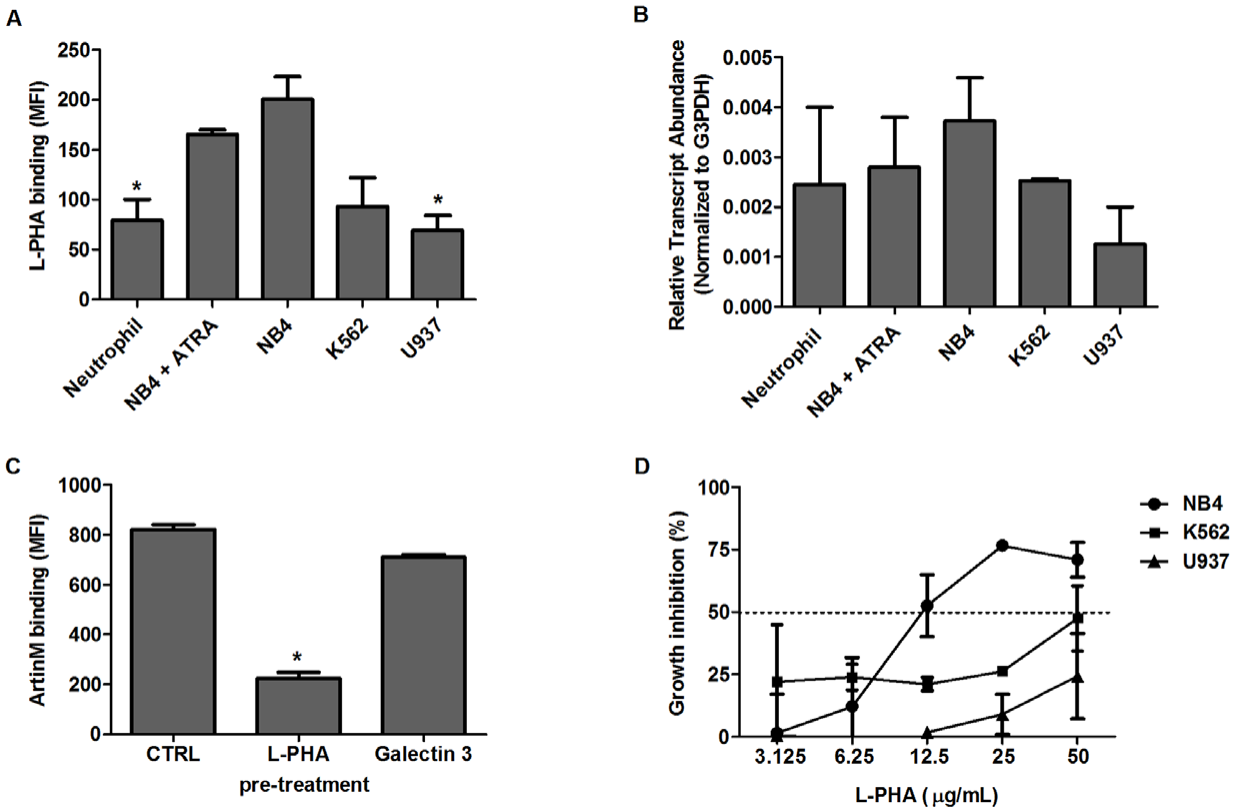
β1-6GlNAc branched N-glycans as targets for lectin recognition and cell growth inhibition. **A**) Binding of β1-6GlNAc by lectin L-PHA in leukemia cell lines: NB4, K562, and U937 cells were incubated with L-PHA-FITC for 30 min (5 µg/mL). Blood neutrophils from healthy donors and NB4 cells pretreated with ATRA were also assayed. Lectin binding was detected by flow cytometry and expressed as mean fluorescence intensity (MFI). **B**) GnT-V mRNA levels in myeloid cell lines: NB4, K562, and U937 cells, as well as blood neutrophils from healthy donors (CD11b^+^) and NB4 pretreated with ATRA (1 µM, 48 h) were assayed. Average Ct values from triplicate samples obtained for each gene, with a standard deviation of less than 0.5 Ct units, were converted to linear values and normalized to the housekeeping gene G3PDH. **C**) L-PHA, but not galectin-3, competes for the ArtinM target on NB4 cells: NB4 cells were incubated with L-PHA or Galectin-3 (5 µg/mL) and then assayed for ArtinM binding (see figure 1). ArtinM binding was detected by flow cytometry and expressed as mean fluorescence intensity (MFI). **D**) Inhibition of leukemia cell growth by L-PHA: NB4, K562, and U937 cells were cultured for 48 h in the presence or absence of 3.125–50 µg/mL L-PHA. Cell growth was evaluated by MTT assay. The curves show the inhibition of cell growth by L-PHA relative to the growth of untreated cells. Each point was obtained from a triplicate assay. The dotted line indicates the L-PHA concentration necessary to inhibit growth by 50% (IC_50_). The result shown are representative of 3 independent experiments and are expressed as mean ± SD, *p<0.05 (Tukey's test).

As ArtinM binding to the core of N-glycans is preserved when a branch is added to Manα1-6 (unpublished glycoarray study results), we tested whether β1,6-GlcNAc branching, which is frequently present in cancer cells [23], was present in leukemia cells. Its occurrence was disclosed by flow cytometry using fluorescent *Phaseolus vulgaris* leukophyto-hemagglutinin (L-PHA). The proportions of L-PHA-stained cells were 98%, 86%, and 69% for NB4, K562, and U937 cells, respectively (data not shown). As shown in Figure 6A, L-PHA staining was conspicuously strongest in NB4 cells, especially in comparison with U937 cells, while intermediate staining was detected in K562 cells. Weak L-PHA staining was also detected in ATRA-differentiated NB4 cells and neutrophils (CD11b^+^ cells) from healthy donors in comparison with that of untreated NB4 cells, indicating a lower incidence of β1,6-GlcNAc branched N-glycans in the non-malignant cells. As expected, the detection of the β1,6-N-acetyl-glucosaminyltransferase (GnT-V or Mgat5) transcript in NB4, K562, U937, neutrophils (CD11b^+^ cells), and ATRA-differentiated NB4 cells was consistent with the incidence of β1,6-GlcNAc-branched N-glycans (Fig. 6B). In addition, L-PHA binding to SW-treated cells was inhibited 80% (data not shown) while ArtinM binding was preserved.

The presence of β1,6-GlcNAc-branched N-glycans recognized by L-PHA on NB4 cells led us to investigate whether this lectin could compete with ArtinM for a related carbohydrate target. As shown in Figure 6C, L-PHA inhibited ArtinM binding to NB4 cells by 75%, suggesting that ArtinM and L-PHA recognize targets that are partially shared. Therefore, we evaluated whether L-PHA inhibits leukemia cell growth in the same manner as ArtinM. As shown in Figure 6D, L-PHA treatment yielded a growth inhibition curve similar to that observed with ArtinM, particularly in NB4 cells, wherein the IC_50_ was 12 (±1) µg/mL. Since galectin-3 recognizes N-acetyllactosamine-containing N-glycans, we also performed a competition binding assay with ArtinM and observed no competition, even after neuraminidase treatment (Fig. 6C). Moreover, galectin-3 did not inhibit NB4 cell growth (data not shown) as did ArtinM and L-PHA. We concluded that lactosamine elongation of the β1,6-GlcNAc branch is irrelevant to ArtinM binding and that lactosamine recognition distant from the N-glycan core does not inhibit leukemia cell growth.

## Discussion

In this study, we demonstrate that ArtinM interaction with NB4 myeloid leukemia cells suppresses cell proliferation. This effect was attributed to the induction of cell death, apparent by the exposure of phosphatidylserine on the cell surface and disruption of mitochondrial membrane potential. Nonetheless, cell death was not accompanied by cell differentiation, caspase-3 activation, or DNA fragmentation. Moreover, augmented ROS production and detection of acidic vesicular organelles in ArtinM-stimulated cells strongly suggests the occurrence of autophagy-associated cell death. The ArtinM carbohydrate recognition domain directly triggers its activities on the cells, where N-glycans on the cell surface glycoproteins are targets for recognition.

Among 3 different leukemia cell lines, NB4 was the most sensitive to ArtinM-induced suppression of cell proliferation. The NB4 cell line has a t(15;17)-positive karyotype and is considered an appropriate model of acute promyelocytic leukemia (APL) for drug evaluation. APL, a specific subtype of acute myelogenous leukemia, is frequently associated with reciprocal translocations between chromosomes 17 and 15 [t(15;17)], leading to fusion of the retinoic acid receptor α (RARα) and promyelocytic leukemia (PML) genes. The PML/RARα fusion product acts as a transcription repressor and blocks the differentiation of APL blasts at the promyelocyte stage [22], [24], [25]. The blockage can be reverted by pharmacological doses of all-trans-retinoic acid (ATRA), constituting the mainstay of APL therapy [25]. The simultaneous administration of ATRA and anthracycline-based chemotherapy is currently considered the standard treatment for newly diagnosed APL patients, leading to high rates of remission. APL relapses are associated with ATRA resistance. Alternative therapy with As_2_O_3_ induces high rates of remission and is being explored as induction treatment. Because it is not associated with myelosuppression and other severe complications associated with anthracycline administration, As_2_O_3_ is used to treat newly diagnosed APL patients in whom chemotherapy is contraindicated. Other therapeutic strategies under development include histone acetylase inhibitors, which revert the PML/RARα transcription repression and potentiate ATRA-induced granulocytic differentiation [26] and granulocyte colony-stimulating factor (G-CSF), which binds to the G-CSF receptor on acute myeloid blasts and reduces their proliferation as a result of increased commitment to terminal differentiation [27].

NB4 cells undergo differentiation by ATRA and apoptosis by As_2_O_3_ treatment, responses that were confirmed in our *in vitro* experiments. The distinct sensitivity of the assayed cell lines to ArtinM was associated with the level of lectin binding to the cell surface. The dependence on recognition of the trimannoside that constitutes the common core structure of N-glycans was demonstrated by (*a*) the inhibition of ArtinM binding to the NB4 cell surface by Manα1-3(Manα1-6)Manβ1 and (*b*) the cell response to ArtinM in TM-treated NB4 cells. Indeed, our previous work on the biological properties of ArtinM showed that its binding to glycosylated receptors on the cell surface is responsible for the triggered responses. This is true in (*i*) human neutrophils, whose CXCR2 glycan recognition by ArtinM accounts for the induction of haptotactic cell migration, increased mediator release, and enhancement of effector functions, such as phagocytosis and respiratory burst [28]; (*ii*) macrophages and dendritic cells, whose TLR2 glycan recognition by ArtinM triggers IL-12 production, which induces Th1-based immunity *in vivo* that confers protection against intracellular pathogens [20]; and (*iii*) mast cell FcεR glycan recognition by ArtinM, which accounts for mast cell recruitment, degranulation, and release of inflammatory mediators [17].

ArtinM-induced NB4 cell death, clearly demonstrated by phosphatidylserine surface exposure and disruption of mitochondrial membrane potential, was not associated with cell maturation, as provoked by other drugs, like ATRA. Indeed, NB4 cell death was caspase-independent and was not accompanied by DNA fragmentation. Moreover, ArtinM treatment induced ROS generation, at levels as high as those provoked by As_2_O_3_. Caspase-independent/ROS-dependent apoptosis can occur through calcium-mediated mitochondrial membrane potential depolarization, leading to the translocation of apoptotic factors such as apoptosis-inducing factor from the mitochondria into the nucleus, which could preserve an apoptotic phenotype in absence of caspase activation [29]. The occurrence of caspase-independent cell death was previously reported in leukemia and lymphoma cells [30]–[32] and can be promoted by abrinA from *Abrus precatorius* [11] and a marine sponge lectin, named CvL [33]. Moreover, the formation of acidic autophagic vacuoles in ArtinM-treated NB4 cells strongly suggests the occurrence of autophagy-associated cell death, which is critical to the antineoplastic response [34], [35]. Autophagy of NB4 cells was previously observed following treatment with platonin or As_2_O_3_ [36]–[38] and is a critical mechanism for induction of the antileukemic effects of arsenic trioxide.

Considering that NB4 responses to ArtinM treatment were relevant and triggered by carbohydrate recognition, the identification of glycans that could be targeted by the lectin was further explored. Among the glycan alterations that occur in malignant transformed cells [1], the most common is increased levels of N-glycans containing the ß1,6-GlcNAc branch linked to the α1,6-mannose of the trimannosyl core [23]. Thus, this type of modification was the focus of our work.

ArtinM CRD is composed of a primary and a secondary site. The primary site interacts with Manα1-3(Manα1-6)Manβ1-4 and the secondary site interacts with other carbohydrates associated with Manα1-3(Manα1-6)Manβ1-4, such as Xylose in horseradish peroxidase [39]. The branch attached to Manα1-6 also contributes to ArtinM recognition, such as a β1,6 branch [40] and (unpublished glycoarray). Despite the preserved binding of ArtinM in swainsonine treated cells, the cytotoxic effect of ArtinM was significantly reduced, which demonstrates how essential the interactions established by the lectin secondary site are for promoting cell death.

**Figure 7:**
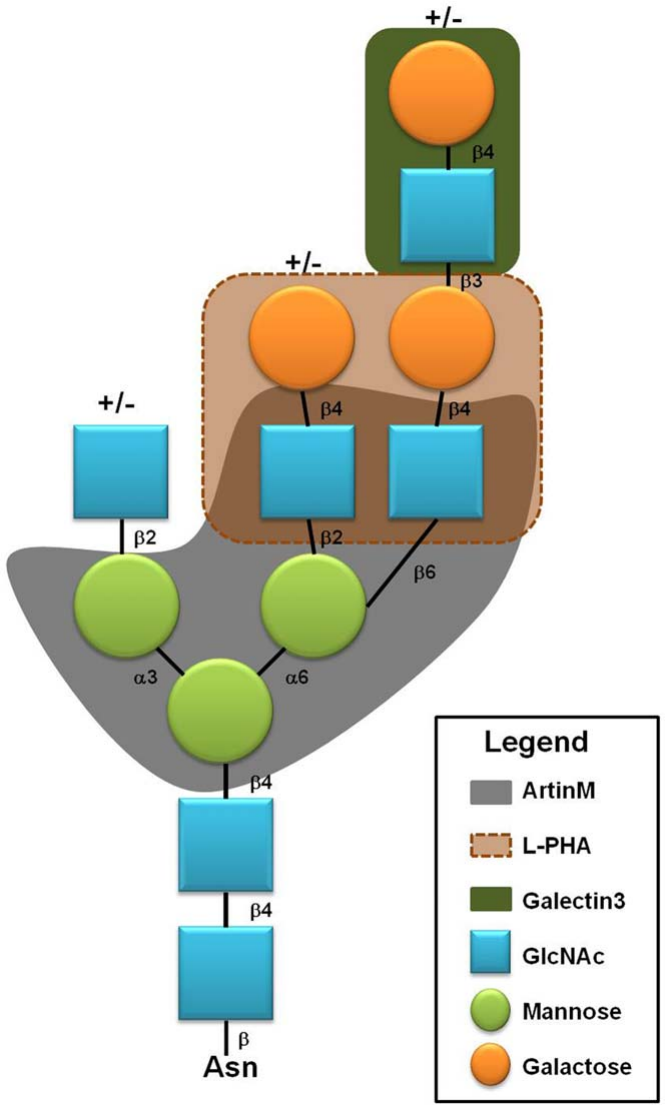
β1-6GlcNAc branched N-glycan is targeted by three different lectins. The targeted areas by ArtinM (grey), L-PHA (brown) and galectin-3 (green) are highlighted in the figure. ArtinM binds to Manα1-3(Manα1-6)Manβ-R core and posses a sub domain that establishes additional interaction with GlcNAc in the context of α1-6Mannose branch. L-PHA binds to the sequence Galβ1-4GlcNAcβ1-2(Galβ1-4GlcNAcβ1-6)Manα-R, which partially merges with the area targeted by ArtinM. Galectin-3 binds to distal poly-N-acetyllactosamines, which does not merge with the areas targeted by ArtinM or L-PHA.

The β1,6 GlcNAc branch of N-glycans on the surface of leukemia cell lines, detected by L-PHA binding, was prevalent on NB4 cells. This result correlated with the level of the N-acetylglucosaminyltransferase V (GnT-V, also named Mgat5) transcript, which was 3 times higher in NB4 cells than in K562 and U937 cells. GnT-V transcription is stimulated by several oncogenes, including *src*, *her-2/neu*, H-*ras*, and v-*sis* [41]–[43] and is downregulated by cell differentiation. This was clearly demonstrated in the HL-60 promyelocytic leukemia cell line, wherein ATRA-induced differentiation was followed by decreased GnT-V activity and a concomitant decrease in N-glycans containing the β-1,6 GlcNAc branch [44]. Because L-PHA competes with ArtinM for binding to NB4 N-glycans and, similar to ArtinM, is able to suppress NB4 cell growth, we hypothesize that recognition of β1,6-GlcNAc-branched N-glycans attached to the protein backbone of still unidentified receptors on NB4 cells accounts, at least partially, for the lectin-induced cell death. Furthermore, β1,6-GlcNAc-branch elongation by N-acetyl polylactosamine is not targeted by ArtinM and is not involved in triggering cell death since galectin-3, a known N-acetyl polylactosamine recognition protein, did not compete with ArtinM for binding to NB4 cells or induce NB4 cell death. The Figure 7 illustrates the carbohydrate target of three different lectins. Our data did not exclude the possibility that other glycan modifications may also be important in triggering ArtinM effects, but certainly establish that β1,6-GlcNAc-branched N-glycan recognition is a significant step in the process.

In summary, ArtinM induces death of NB4 cells by an autophagic-associated pathway and by recognition of Manα1-3(Manα1-6)Manβ1 in the context of β1,6-GlcNAc-branched N-glycans. ArtinM acts as a very potent suppressor of cell growth, offering a novel potential strategy for leukemia therapy. An additional advantage of ArtinM as a therapeutic agent is its immunomodulatory property, responsible for the induction of Th1 immunity, a response that is potentially effective against leukemia progress. Finally, the fact that 2 different lectins, both specific for β1,6-GlcNAc-branched N-glycans, are able to suppress leukemia cell growth provides insights into its potential as a new target for anti-leukemia treatment.

## Materials and Methods

### Cells

Leukemia cell lines (K562 [45], NB4 [46], and U937 [47]) were cultured in RPMI 1640 supplemented with 10% heat-inactivated fetal bovine serum, streptomycin/gentamycin (100 µg/mL) (Gibco) and incubated at 37°C in a humidified atmosphere containing 5% CO_2_.

Heparinized human blood from healthy volunteers was layered on a density gradient medium for neutrophil isolation (Mono-poly, density 1,114, MP Biomedical) and centrifuged at 400×*g* for 30 min. Neutrophils were washed by centrifugation and suspended in RPMI medium at 10^6^ cells/mL. Samples were subjected to hypotonic lysis to eliminate remaining erythrocytes. Resulting preparations were 98% pure (CD11b^+^), with viability of at least 95%, as measured by trypan blue. Informed written consent from all participants and the study was approved by the Ethics Committes and the Institutional Review Board of the Clinical Hospital of Ribeirão Preto, University of São Paulo [10012/2009 and 10229/2006].

### ArtinM affinity purification

ArtinM, a d-mannose-binding lectin extracted from jackfruit seeds (*Artocarpus heterophyllus* Lam. Syn. *A. integrifolia* L.f.), was purified by affinity-chromatography as previously described [48]. The protein content was measured by BCA kit (Sigma).

### MTT assay

Cells (2×10^4^ cells/mL) were cultured in the presence of 0 to 100 µg/mL ArtinM or L-PHA (*Phaseolus vulgaris* leukophyto-hemagglutinin) for 48 h in 96-well plates. MTT solution was added to the wells at a final concentration of 500 µg/mL. After 3 h incubation, 50 µL DMSO was added into the wells. Cell number and viability were evaluated as previously described [49]. Growth inhibition was calculated: Growth inhibitory rate = (average OD value in the control group – average OD value in the treatment group)/average OD value in the control group×100%. IC_50_ was determined by using a nonlinear regression curve.

### Assessment of apoptosis by Annexin V

Apoptotic cell death was examined by staining with FITC-labeled Annexin V and propidium iodide (PI) (Sigma). Annexin V binds to externalized phosphatidylserine, whereas PI penetrates the increasingly permeable plasma membrane during necrosis or later stages of apoptosis and binds to cellular DNA. Leukemia cell lines (NB4, U937, and K562) were treated with ArtinM at NB4 IC_50_ (10 µg/mL) for 48 h. Non-treated and ArtinM-treated cells were analyzed for Annexin V and PI staining by flow cytometry (BD FACSCalibur).

### Determination of mitochondrial membrane potential (mΔΨ)

Apoptosis was investigated further by analyzing the mitochondrial membrane potential by JC-1 assay according to the manufacturer's protocol (Cell Technology). Leukemia cell lines (NB4, U937, and K562) were treated with ArtinM at NB4 IC_50_ (10 µg/mL) for 48 h prior to the addition of JC-1 for 30 min. Non-treated and ArtinM-treated cells were analyzed by flow cytometry (BD FACSCalibur). JC-1 dimers appear as red fluorescence in stable mitochondria whereas monomers appear as green fluorescence when the mitochondrial membrane potential decreases.

### Assessment of differentiation by flow cytometry and morphology

The expression of differentiation markers CD14 and CD11b was determined by flow cytometry. NB4 cells were harvested after 48 h incubation with ArtinM (10 µg/mL), washed twice with PBS, and then incubated for 30 min at room temperature with mouse anti-human PE-conjugated CD11b mAb (BD Bioscience) and mouse anti-human FITC-conjugated CD14 mAb (BD Bioscience). Mouse isotypes matching IgGs were used to set threshold parameters for flow cytometry. In addition, cytospin preparations stained with HEMA 3 (Biochemical Sciences) were used for morphological evaluation. Cells treated with ATRA (1 µM) were used as a positive control.

### Accumulation of ROS (Reactive Oxygen Species)

NB4 cells (2×10^4^ cells/mL) were cultured in the presence of ArtinM (10 µg/mL) for 48 h in 96-well plates. ROS levels were determined by measuring the oxidative conversion of cell-permeable 2′,7′ dichlorofluorescein diacetate (DCFH-DA, Sigma), after incubation for 30 min at 37°C, to fluorescent dichlorofluorescein (DCF) in a fluorospectro-photometer with excitation and emission wavelengths of 485 and 535 nm, respectively. Cells treated with As_2_O_3_ (1 µM) were used as a positive control. Cells were also co-treated with ArtinM and antioxidants: α-tocopherol acetate (10 µM, Sigma) or reduced glutathione (50 µM, Sigma).

### Western blot analysis of caspase 3

RIPA cell lysates (100 µg) were electrophoresed on a 12% SDS-polyacrylamide gel (Bio-Rad) and electroblotted to a nitrocellulose membrane (Millipore Corp). The membrane was incubated overnight with anti-caspase 3 antibody (1 µg/mL, R&D Systems) followed by a secondary horseradish peroxidase-conjugated anti-mouse antibody (Amersham Biosciences). Detection was performed with SuperSignal® chemiluminescence substrate (Pierce). Blots were incubated overnight with murine monoclonal G3PDH antibody (R&D, 1∶2000 dilution) followed by a secondary horseradish peroxidase-conjugated sheep anti-rabbit antibody (Amersham Biosciences). Cells treated with staurosporine (5 µM, 4 h, Sigma) were used as a positive control.

### Electrophoretic analysis of DNA fragmentation

DNA in the lysates of NB4 cells cultured in the presence of ArtinM was extracted with a DNA Purification Kit (Promega) and fragmentation was visualized after electrophoresis on 1% agarose gels containing 0.5 mg/mL ethidium bromide and photographed with a Bio-Rad GD2000 (Bio-Rad). Cells treated with staurosporine (5 µM, 4 h) were used as a positive control.

### NB4 treatment with tunicamycin (TM) and swainsonine (SW)

NB4 cells (1×10^6^ cells/mL) were cultured in the presence of 5 µg/mL TM or SW(Sigma) for 24 h. Cells were washed twice with PBS and incubated for 30 min at 4°C with FITC-conjugated ConA or biothyl-ArtinM/streptavidin-FITC. Cells were tested for positive staining using flow cytometry. TM- or SW-treated cells were incubated in the presence of ArtinM for 48 h. Growth inhibition was measured by MTT.

### Detection of acidic vesicular organelles with acridine orange staining

To quantify the development of acidic vesicular organelles (AVOs), ArtinM-treated cells were stained with acridine orange (10 µg/mL) for 15 min and visualized by confocal laser scanning microscopy (Leica SP5, Leica Microsystem, Wetziar, Germany). In cells stained with acridine orange, the cytoplasm and nucleoli emit green fluorescence while the acidic compartments emit red fluorescence, the intensity of which is proportional to the acidity.

### ArtinM and L-PHA binding and competition binding assay

Cells were fixed with 2% paraformaldehyde at room temperature for 20 min and incubated with biotinylated ArtinM/streptavidin-FITC (5 µg/mL in PBS) or L-PHA-FITC (*Phaseolus vulgaris* leukophyto-hemagglutinin) for 30 min. Lectin binding was measured by flow cytometry. NB4 cells were fixed with 2% paraformaldehyde at room temperature for 20 min and then incubated with L-PHA (5 µg/mL) for 10 min. Finally, cells were incubated with biotinylated ArtinM/streptavidin-FITC (5 µg/mL). The competition binding analysis was performed by flow cytometry.

### Fluorescence microscopy

NB4 cells were placed on coverslips coated with Biobond, incubated with biotinylated ArtinM (5 µg/mL in PBS) or with PBS alone, at 4°C for 60 min, and fixed with 2% paraformaldehyde at room temperature for 20 min. For some assays, ArtinM was pre-incubated with 200 mM of d-Galactose or 10 µM of Manα1-3[Manα1-6]Man, for 60 min at room temperature. Cells were rinsed and then incubated with streptavidin-FITC for 30 min. Coverslips were mounted with Fluoromount-G and examined by fluorescence microscopy (Axiophot, Carl Zeiss AG, Germany).

### Real Time PCR for Mgat5

Total RNA isolation was performed using Trizol Reagent (Life Technologies, Inc., Gaithersburg, MD, USA), as indicated by the manufacturer. cDNA synthesis was performed in a final volume of 20 µL, using ImProm-II Reverse Transcriptase (Promega Corporation, Madison, WI, USA). The reaction mixture contained 4 µg total RNA, 20 pmol oligo dT primer (Life Technologies), 40 U RNasin, 500 µM dNTP mix, and 1 U reverse transcriptase in 1× reverse transcriptase buffer. The cDNA was treated with 10 µg RNase (Gibco) and immediately used or stored at −20°C. PCR amplification and analysis were performed on an ABI Prism 7500 sequence detector (Applied Biosystems, Foster City, CA, USA). All reactions were performed with SYBR Green Master Mix (Applied Biosystems) in 25 µL reaction volumes containing 2 µL template cDNA, 5 pmol of each primer, and 12.5 µL SYBR Green (Applied Biosystems). The primers for PCR amplification were:

R: 5′-TGAGTTCGCTGCTGGATGGT-3′
F: 5′-TCACTCCGTGGAAGTTGTCCT-3′

Triplicate Ct values for each gene were averaged, and the standard deviation was calculated. Samples that resulted in a standard deviation of >0.5 Ct units were rerun until values with standard deviations within an acceptable range were acquired. The logarithmic average Ct value for each gene and the control gene was converted to a linear value using the conversion: 2 - Ct. Converted values were normalized to G3PDH by dividing the individual gene value by the control gene value. Normalized values were scaled.

### Statistical analysis

Results are presented as mean ± SD and all statistical analyses were calculated with Prism (Graph Pad Software). Comparisons between groups were done by analysis of variance, followed by Tukey's test or Student's t test. The level of statistical significance was p<0.05.
